# Inhibition of* Pseudomonas aeruginosa* Biofilm Formation by Traditional Chinese Medicinal Herb* Herba patriniae*

**DOI:** 10.1155/2017/9584703

**Published:** 2017-03-09

**Authors:** Bo Fu, Qiaolian Wu, Minyan Dang, Dangdang Bai, Qiao Guo, Lixin Shen, Kangmin Duan

**Affiliations:** ^1^Key Laboratory of Resources Biology and Biotechnology in Western China, Ministry of Education, College of Life Sciences, Northwest University, Xi'an, Shaanxi 710069, China; ^2^Department of Oral Biology & Medical Microbiology, Rady Faculty of Health Sciences, University of Manitoba, 780 Bannatyne Ave., Winnipeg, MB, Canada R3E 0W2

## Abstract

New antimicrobial agents are urgently needed to treat infections caused by drug-resistant pathogens and by pathogens capable of persisting in biofilms. The aim of this study was to identify traditional Chinese herbs that could inhibit biofilm formation of* Pseudomonas aeruginosa*, an important human pathogen that causes serious and difficult-to-treat infections in humans. A* luxCDABE*-based reporter system was constructed to monitor the expression of six key biofilm-associated genes in* P. aeruginosa*. The reporters were used to screen a library of 36 herb extracts for inhibitory properties against these genes. The results obtained indicated that the extract of* Herba patriniae *displayed significant inhibitory effect on almost all of these biofilm-associated genes. Quantitative analysis showed that* H. patriniae *extract was able to significantly reduce the biofilm formation and dramatically altered the structure of the mature biofilms of* P. aeruginosa*. Further studies showed* H. patriniae *extract decreased exopolysaccharide production by* P. aeruginosa* and promoted its swarming motility, two features disparately associated with biofilm formation. These results provided a potential mechanism for the use of* H. patriniae *to treat bacterial infections by traditional Chinese medicines and revealed a promising candidate for exploration of new drugs against* P. aeruginosa* biofilm-associated infections.

## 1. Introduction

*Pseudomonas aeruginosa* is a remarkably adaptive bacterial pathogen which can cause persistent infections in burn patients, immune-compromised patients, and individuals with the genetic disease cystic fibrosis. It is one of the most prevalent nosocomial pathogens, and the infections caused by* P. aeruginosa* can be very serious and life-threatening [1].

Biofilm formation is a major characteristic of* P. aeruginosa *chronic infections [2, 3].* P. aeruginosa *cells in biofilms are surrounded by exopolysaccharides and form a structured aggregates, and these cells exhibit increased resistance to antibiotics and other adversary agents [3–6]. Infections caused by biofilm-forming* P. aeruginosa, *such as those in cystic fibrosis lung, are almost impossible to eradicate [7]. There is an urgent need to find novel antimicrobial agents to control such infections [8, 9].

Traditional Chinese medicines (TCMs) have been widely used to treat infectious diseases for more than a thousand years in China. Many components constituents of TCMs have been found to be very effective in treating bacterial infections such as gastritis, stomatitis, dermatitis, and bacterial pneumonia [10]. However, the mechanisms of these herbs in treatment infectious diseases are mostly unknown.

*Herba patriniae *is a perennial herbal of TCM, which contains various beneficial ingredients such as amino acids, vitamins, minerals, alkaloids, tannins, and saponins. It has been reported to have functions of antioxidant, antibacterial, antiviral, blood-activating and stasis-eliminating, promoting regeneration of liver cells, and anxiety-alleviating. The boiling water extracts of* H. patriniae *had been identified having anticyanobacteria activity against* Microcystis aeruginosa *[11].

In this study, 36 extracts of 18 Chinese herbs that are commonly used for treating infection-like symptoms were screened for inhibitory effect against* P. aeruginosa* biofilms. We found that the extract of the Chinese herb* H. patriniae *significantly inhibited the expression of the genes associated with biofilm formation in* P. aeruginosa* PAO1. It reduced exopolysaccharide production and biofilm formation and then altered the structure of the mature biofilms.

## 2. Materials and Methods

### 2.1. Bacterial Strains and Culture Conditions

Bacterial strains used in this study are listed in Table 1. All the strains were cultured in LB (Luria Bertani) broth at 37°C with orbital shaking at 200 rpm or on LB agar plates supplemented with antibiotic of kanamycin (Kan, 50 *μ*g/mL) or trimethoprim (Tmp, 300 *μ*g/mL) where appropriate.

### 2.2. Traditional Chinese Medicine Extraction

Traditional Chinese medicinal herbs were selected according to their efficacy in treatment of infection-like symptoms in Chinese medicine. They were obtained from local pharmacy (Yikang Pharm chain store, China). The pulverized herb was firstly immerged in 75% ethanol or deionized water, respectively, for 2 h and then boiled for 2 h additionally (weight to solvent volume was 1 : 5). The extracts were filtered by filter paper and were evaporated under vacuum at 35°C using a rotary evaporator (Buchi, Switzerland). The concentrated extracts were then freeze-dried using a lyophilizer and stored at −80°C. The water extracts were redissolved in deionized water and ethanol extracts were redissolved in methanol and then sterilized immediately with 0.22 *μ*m Iwaki filter before use.

### 2.3. Gene Expression Assay

Key genes* algU *[12],* pslM *[13, 14],* pelA *[15],* algA *[16],* ppyR *[17], and* bdlA *[18] that are known to be involved in biofilm formation in* P. aeruginosa* were selected to construct* luxCDABE*-based promoter-reporter fusions (Table 2). The reporters were constructed as previously described [19, 20] and were subsequently transformed into PAO1. Using* luxCDABE*-based reporters, gene expression in liquid cultures was measured by light luminescence (in counts per second) in a Victor^3^ multilabel plate reader (Perkin-Elmer). Strains containing different reporters were cultivated overnight in LB broth supplemented with Tmp (300 *μ*g/mL) and then diluted to an optical density at 600 nm of 0.2. The diluted cultures were used as inoculants. After additional 2 h incubation, 5 *μ*L cultures were inoculated into parallel wells on a 96-well black plate containing 93 *μ*L medium and 2 *μ*L herbal extract in different concentrations. 50 *μ*L mineral oil was added to the wells to prevent evaporation. Promoter activities were measured every 30 min for 24 h in a Victor multilabel plate reader and bacterial growth was monitored by measuring OD_600_ at the same time.

### 2.4. Gene Expression Measurement by Real-Time Quantitative PCR (RT-qPCR)

Bacterial RNA was extracted using RNAprep Pure Cell/Bacteria Kit (TIANGEN). 1 *μ*g of RNA sample was reverse transcribed to cDNA using 1st Strand cDNA Synthesis Kit (Takara). Real-time qPCR was performed using SuperReal PerMix Plus (SYBR Green/probe) (TIANGEN) and primers specific for* algU* (Forward: 5′-AACACCGCGAAGAACCACCT-3′, Reverse: 5′-ATCTCATCCCGCAACATCGCG-3′),* algA *(Forward: 5′-AGAACTGAAGAAGCACGACG-3′, Reverse: 5′-TTCTCCATCACCGCGTAGT-3′),* pelA *(Forward: 5′-ATGGCTGAAGGTATGGCTG-3′, Reverse: 5′-AGGTGCTGGAGGACTTCATC-3′), and* pslM* (Forward: 5′-CTATGACGCACGGCAACTGG-3′, reverse: 5′-CGCCATTGACCAGGTGCAT-3′). The values obtained were normalized to the housekeeping gene* proC* (Forward: 5′-CAGGCCGGGCAGTTGCTGTC-3′, reverse: 5′-GGTCAGGCGCGAGGCTGTCT -3′).

### 2.5. Quantification of the Biofilm Formation

Biofilm formation was measured in 96-well polystyrene microtiter plate as described previously with minor modifications [21]. 10 *μ*L of overnight cultures of PAO1 was added to 90 *μ*L fresh LB broth containing 160 *μ*g* H. patriniae *water extract. Same volume of deionized water was used as control. After being cultured with shaking at 200 rpm for 1 h the plates were kept at stationary state at 37°C for 3 days and 7 days with a medium replacement at every 24 h interval. Then wells were washed twice with deionized water gently to remove the planktonic cells. The sedentary cells were stained with 1% (v/v) crystal violet solution for 15 min. Unbound dye in the wells were washed off by deionized water before 200 *μ*L of 95% ethanol was added to dissolve the crystal violet stains. The absorbance of the solutions was then measured at 570 nm [7, 22].

### 2.6. Biofilm Imaging by Silver Staining Method

To examine the biofilm structure, silver staining method was used as described previously with some modifications [23, 24]. Similar to the above described biofilm assay, biofilms were grown on cover slip (8 mm diameter) placed at bottom of the wells of 24-well plates. 200 *μ*L of fresh bacterial culture was inoculated to 1.8 mL TSB supplemented with 3.2 mg* H. patriniae*. The plates were incubated at 37°C with TSB medium replaced every 24 h. After 3 days or 7 days of cultivation, the cover slip was taken out and washed three times with saline water to remove the planktonic cells. The cover slip was immersed in 2.5% glutaraldehyde for 1 h for the cells fixation and rinsed with distilled water for 1 min. Then the cover slip was immersed in saturated calcium chloride solution for 15 min and rinsed with distilled water for 5 min. The biofilms on the cover slip were then stained with 5% silver nitrate for 15 min, followed by 1% hydroquinone colour-rendering for 2 min and then rinsed with distilled water for 1 min. Fixation was treated with 5% sodium thiosulfate for 1 min, followed by a final water rinse. The cover slip was placed on an inverted optical microscope for biofilm structure observation.

### 2.7. Swarming Motility Assay

Swarming assay was carried out as previously reported [25]. The medium used for swarming motility assay consists of nutrient broth (0.8%), glucose (0.5%), and agar (0.5%). The plates were dried at room temperature overnight before being used. 2 *μ*L of* P. aeruginosa* PAO1 culture (OD600 = 0.5) mixed with 106.67 *μ*g of* H. patriniae* water extract was spotted onto the swarming plate and the one with deionized water was used as blank control. Plates were incubated at 37°C for 24 h before the swarming diameter was measured.

### 2.8. Measurement of Exopolysaccharide Production

Overnight culture of PAO1 (OD600 = 0.005) was spotted onto Congo red plates (1% Tryptone, 1% agar, 4% Congo red, and 2% Coomassie blue) with or without* H. patriniae* water extract. The amount of* H. patriniae* water extract was 64 *μ*g. The colony morphology and staining were observed after 3 days of incubation at 37°C [22].

## 3. Results and Discussion

### 3.1. Screening for Herbs with Inhibitory Effect on* P. aeruginosa* Biofilm-Associated Genes

**Figure 1:**
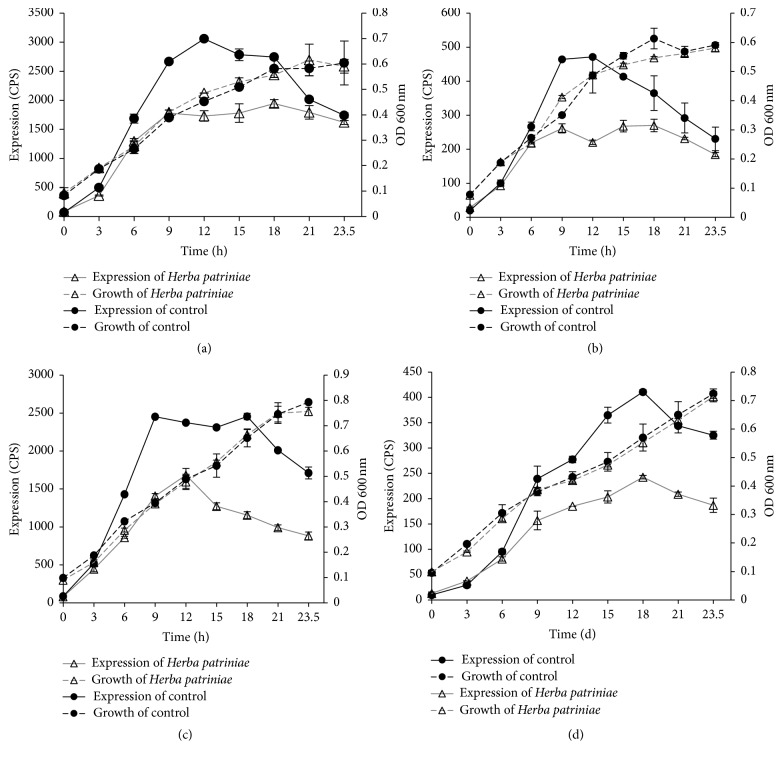
Expressions of* algU*,* algA*,* pslM,* and* bdlA* in medium with water extract of* Herba patriniae.* Expressions of* algU* (a),* algA* (b),* pslM *(c), and* bdlA* (d) in medium (total volume of 100 *μ*L) with 160 *μ*g* H. patriniae *extract and the controls were the ones without herbal extract.

Eighteen traditional Chinese medicinal herbs were selected because of their common usage for infection-like symptoms. Both boiling water extracts and ethanol extracts were obtained and used to screen for antibiofilm activities. Since* P. aeruginosa* biofilm formation is directly associated with the activity of several known genes, we constructed* luxCDABE*-based reporters to examine the effect of herb extracts on these genes. The effects of the crude extracts on the expression of these biofilm-associated genes (*algU*,* pslM*,* pelA*,* algA*,* ppyR*, and* bdlA*) are presented in Table 3. The results indicate that different herbal extracts exhibited various degrees of inhibitory effects on these genes. The water extract of* H. patriniae* showed the most significant effect on the expression of* algU*,* algA*,* pslM*, and* bdlA*. Examples of the gene expression profiles in the presence of* H. patriniae* are shown in Figure 1.

To confirm the results obtained from the* lux*-based reporter assay, real-time qPCR was carried out using bacterial RNA samples isolated in the presence and absence of* H. patriniae*. The results are shown in Table 4. In agreement with the results from the reporter assay, the mRNA levels of* algU*,* algA*,* pslM*, and* bdlA* in PAO1 were all significantly decreased in the presence of* H. patriniae* extract (at 1.6 mg/mL) compared with those in the absence of* H. patriniae* extract. It is noted that the inhibition of* algU* and* algA* was more pronounced in the qPCR assay.

The results indicate that many of these herbs have an inhibitory effect on the genes associated with biofilm formation in* P. aeruginosa*. This is somewhat not surprising because they all have been used for treatment of chronic bacterial infections in traditional Chinese medicine.

The effect from the water extract of* H. patriniae *was remarkable as it inhibited five genes tested. It has been reported that the herb had antibacterial and antiviral activity. However, it was noted that the water extract did not inhibit the growth of* P. aeruginosa* (Figure 1). Even though conventional antibiotic compounds may exist in* H. patriniae *against other bacteria, this result indicates no such component was present against* P. aeruginosa*, at least not at the concentrations used in our experiments.

Lacking of bacterial killing or growth inhibition activity, however, may not be a weakness of such herbs in treating infectious diseases. As discussed in previous reports [26–29], a promising new class of antipathogenic drugs that target virulence factors and/or biofilm formation instead of killing the pathogens has many advantages in clinical use. First, these antipathogenic drugs theoretically are less likely to render drug resistance in the pathogens because they do not assert a selective pressure on the pathogen's survival. Second, such therapeutics would unlikely affect other nonpathogenic or beneficial bacteria, that is, the microbiome in the host.

### 3.2. Extract of* H. patriniae* Inhibits* P. aeruginosa* Biofilm Formation

**Figure 2:**
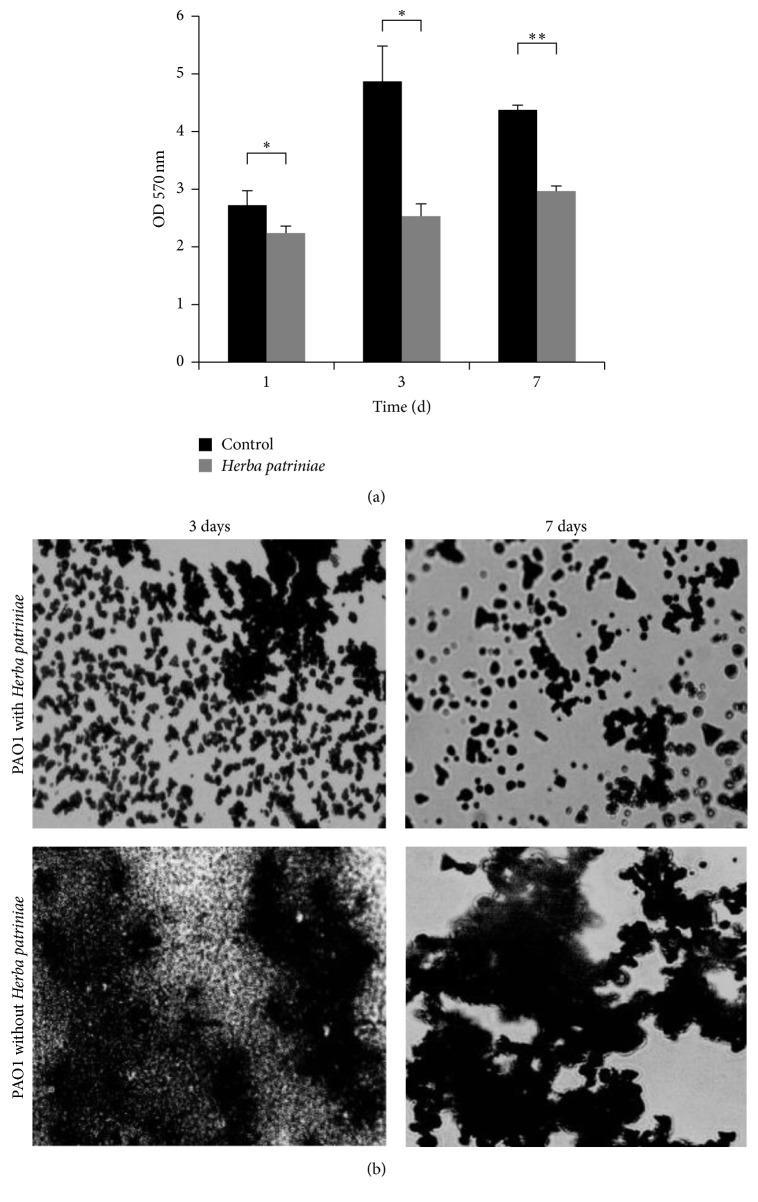
(a) Inhibition of biofilm production of PAO1 by* H. patriniae *extract. The control did not contain any herb extract. “*∗*” indicates significant difference between the herb group and the control group (*P* < 0.05). “*∗∗*” indicates very significant difference (*P* < 0.01). (b) Micrographs of biofilms formed with and without* H. patriniae*. Top row, PAO1 silver stained biofilms in the presence of extract of* H. patriniae *after 3 days and 7 days of incubation. Bottom row, PAO1 silver stained biofilms without* H. patriniae*.

From the gene expression results, the extract of* H. patriniae *presumably would inhibit the biofilm formation of* P. aeruginosa*. To verify such an effect,* P. aeruginosa* biofilm formation was compared in the presence and absence of* H. patriniae *extract. As shown in Figure 2(a), significantly less biofilm was formed in the presence of the herb extract than that without the extract at the irreversible attachment stage (1 d) and mature stage (3 d and 7 d) after inoculation. The result is in agreement with the gene expression data, suggesting the* H. patriniae *extract could reduce biofilm formation through inhibiting the genes associated with biofilm formation.

Importantly, the addition of the* H. patriniae *also dramatically altered the structure of the biofilms (Figure 2(b)). It appears that the herb extract prevented the formation of mature biofilms, only allowing* P. aeruginosa *to form smaller cell clusters. These results indicate that the water extract of* H. patriniae *indeed was able to inhibit* P. aeruginosa* biofilm formation.

### 3.3. Water Extract of* H. patriniae* Inhibited* P. aeruginosa* Exopolysaccharide Production

**Figure 3:**
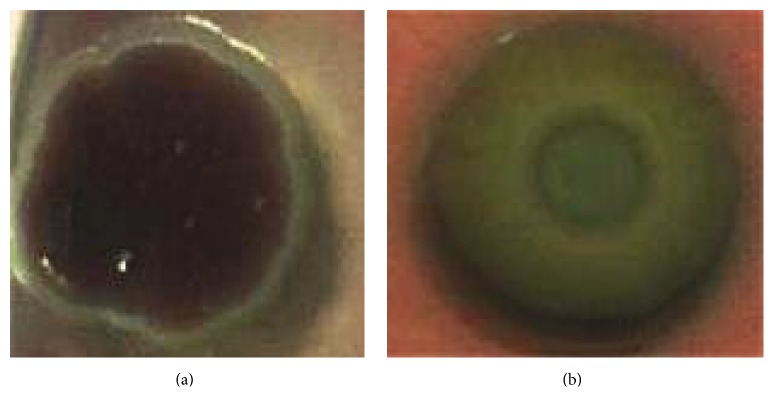
Photograph of exopolysaccharide production on Congo red plates. (a) Without* H. patriniae*. (b) With* H. patriniae*. The colony morphology and staining were observed after 3 days of incubation at 37°C.

The exopolysaccharide (EPS) matrix is an important component of biofilm structure [30]. We compared the exopolysaccharide production in the presence of* H. patriniae *extract to those without* H. patriniae *extract. As shown in Figure 3,* P. aeruginosa* PAO1 cells produced more EPS shown in red by Congo red staining than the cells with* H. patriniae *extract. This result indicates that* H. patriniae *inhibited* P. aeruginosa* exopolysaccharide production and hence affected biofilm formation.

### 3.4. *H. patriniae* Water Extract Promoted PAO1 Swarming Motility

**Figure 4:**
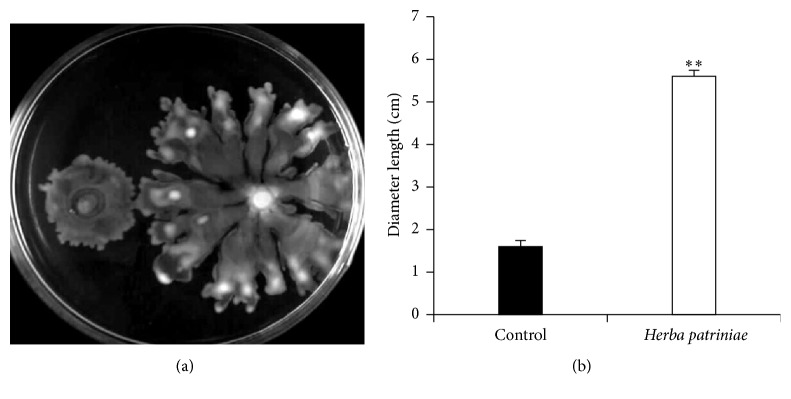
Swarming motility of PAO1. (a) PAO1 grown with* H. patriniae *was at right; the one without herb was at left. (b) The swarming diameter of PAO1 with* H. patriniae*, the control was the one without herb. “*∗∗*” indicates significant difference (*P* < 0.01).

Upon encountering a surface, the surface-associated behaviors of* P. aeruginosa,* such as biofilm formation and swarming, are often coregulated [31]. In* P. aeruginosa*, swarming motility is reversely correlated with biofilm formation. Examination of the swarming motility of PAO1 in the presence and absence of the water extract of* H. patriniae *showed that* H. patriniae *promoted* P. aeruginosa* swarming motility (Figure 4(a)). The diameter of PAO1 grown with 106.67 *μ*g* H. patriniae *was almost 5.60 cm, while the control was less than 2.0 cm (Figure 4(b)). Considering the reverse relationship between swarming motility and biofilm formation, the enhanced swarming motility by* H. patriniae* extract could have contributed the inhibition of biofilm formation.

**Figure 5:**
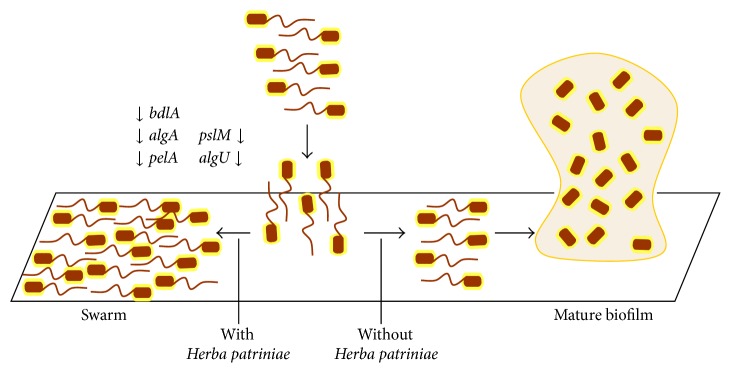
*P. aeruginosa* biofilm formation and swarming motility in the influence of* H. patriniae*.

Taken together, the extract of* H. patriniae *clearly inhibited the biofilm formation of* P. aeruginosa*. It inhibited several key genes* algU*,* pslM*,* pelA*,* algA*, and* bdlA* that are involved in biofilm formation.* H. patriniae *reduced exopolysaccharide production and promoted swarming motility. Increased motility may reduce adhesion and enable bacteria to actively escape the biofilm matrix to become planktonic bacteria [13, 31, 32]. As depicted in Figure 5, multiple factors/pathways probably have contributed to the reduction of biofilm formation and the altered biofilm structure in the presence of* H. patriniae*.

In a time of resistance to multiple antimicrobial agents in pathogenic bacteria being spread, there is an urgent need to develop new antibacterial agents [1, 32]. Drugs against infections that involve biofilms are particularly required. Pathogens in biofilm formation are more resistant to conventional antibiotics and other adversary conditions such as nutritional stress. Biofilms also protect bacterial cells from the activity of host immune response [33, 34].

Traditional Chinese medicines are a valuable source for novel antibacterial agents [35–37]. The inhibitory effect of the water extract of* H. patriniae *against* P. aeruginosa* biofilms and biofilm-related phenotypes signifies that* H. patriniae *is a promising candidate for treatment of infections caused by* P. aeruginosa* biofilms. It could be used in the way of traditional Chinese medicine or it can be explored for active compounds.

## 4. Conclusions

Our results indicate* H. patriniae *extract could significantly inhibit the expression of* P. aeruginosa* genes associated with biofilm formation, alter the structure, and prevent the formation of mature biofilms. It also decreased exopolysaccharide production and promoted swarming motility. These results provided a potential underlying mechanism for the use of* H. patriniae *to treat bacterial infections in traditional Chinese medicine and revealed a promising candidate for exploration of new drugs against* P. aeruginosa* biofilm-associated infections.

## Figures and Tables

**Table 1:** Bacterial strains used in this study.

Strains/plasmids	Description	Source
*E. coli *DH10B	F-mcrA(mrr-hsdRMS-mcrBC)80dlacZ ΔM15ΔlacX74 deoR recA1 endA1 araD139 Δ(ara leu)7697 galU galK*λ*-rpsl nupG	Invitrogen
*P. aeruginosa* PAO1	Wild type	This lab
pMS402	Expression reporter plasmid carrying the promoterless *luxCDABE*; Kan^r^, Tmp^r^	This lab
pKD-*pslM*	pMS402 containing *pslM* promoter region; Kan^r^, Tmp^r^	This study
pKD-*pelA*	pMS402 containing *pelA* promoter region; Kan^r^, Tmp^r^	This study
pKD-*algU*	pMS402 containing *algU* promoter region; Kan^r^, Tmp^r^	This study
pKD-*ppyR*	pMS402 containing *ppyR* promoter region; Kan^r^, Tmp^r^	This study
pKD-*algA*	pMS402 containing *algA* promoter region; Kan^r^, Tmp^r^	This study
pKD-*bdlA*	pMS402 containing* bdlA* promoter region; Kan^r^, Tmp^r^	This study

**Table 2:** Reporter genes and primer sequences used.

PA number	Gene	Function	Primer	Sequence (5′ → 3′)
PA0762	*algU *	RNA polymerase sigma factor	pKD-*algU*-S	GCACTCGAGAGGATGCCTGAAGACCTC
			pKD-*algU*-A	GTAGGATCCGATGGCGATCCGATACAG
PA2243	*pslM*	Succinate dehydrogenase; fumarate reductase flavoprotein	pKD-*pslM*-S	ATCCTCGAGCGGTGCGCAAGAAGACC
			pKD-*pslM*-A	GTTGGATCCCGTAACGCTCGCCCAGTT
PA3064	*pelA*	Glycoside hydrolase; deacetylase	pKD-*pelA*-S	CGTCTCGAGCTTTCCACTTTGCCACAG
			pKD-*pelA*-A	TACGGATCCTACCAGAACGCCACGCT
PA3551	*algA*	Phosphomannose isomerase; guanosine 5′-diphospho-D-mannose pyrophosphorylase	pKD-*algA*-S	TGACTCGAGTGAAGGCGGACTGAGAC
			pKD-*algA*-A	TATGGATCCGCGACCAGTTTCATC
PA2663	*ppyR*	psl and pyoverdine operon regulator	pKD-*ppyR*-S	GCACTCGAGCACTTCTTCTGCTACAGC
			pKD-*ppyR*-A	GTAGGATCCAGCACTTGCACAGCAGAC
PA1423	*bdlA*	Biofilm dispersion locus A	pKD-*bdlA*-S	GCACTCGAGCGTCATATTTCCGACGAA
			pKD-*bdlA*-A	TCAGGATCCGTAGTCTTCCGATTGCG

**Table 3:** Effect of water and ethanolic herb extracts on biofilm-associated genes expression.

The English name of herbs	Water extract						The English name of herbs	Ethanolic extract					
	*algU*	*pslM*	*pelA*	*algA*	*ppyR*	*bdlA*		*algU*	*pslM*	*pelA*	*algA*	*ppyR*	*bdlA*
*Atractylodes lancea*	+1.5	0	−2.5	0	0	0	*Atractylodes lancea*	0	0	0	0	0	0
*Cortex fraxini*	0	−3	−1.5	−1	0	0	*Cortex fraxini*	−1.8	−2.5	−0.5	−1.5	0	0
*Cyrtomium fortunei*	0	−1.5	−2	−1	0	0	*Cyrtomium fortunei*	0	0	0	0	0	0
*Erodium stephanianum *Willd.	a	a	a	a	a	a	*Erodium stephanianum willd*	a	a	a	a	a	a
*Folium artemisiae Argyi*	−2	0	−2	0	−1.5	0	*Folium artemisiae argyi*	0	0	−2	0	−1.5	0
*Fructus quisqualis*	a	a	a	a	a	a	*Fructus quisqualis*	a	a	a	a	a	a
*Herba agrimoniae*	a	a	a	a	a	a	*Herba agrimoniae*	a	a	a	a	a	a
*Herba patriniae*	−2	−2.5	−0.5	−2.5	0	−2	*Herba patriniae*	0	0	0	0	−1.5	−1
*Herba scutellariae barbatae*	0	0	0	−1	0	0	*Herba scutellariae barbatae*	0	0	0	0	0	−1.5
*Pomegranate rind*	−1.5	−1.2	0	−1	0	0	*Pomegranate rind*	−1.5	−1	−1	−0.5	0	0
*Portulacae herba*	+1	0	0	+1.5	0	0	*Portulacae herba*	0	0	0	0	0	0
*Radix paeoniae*	−0.5	−0.5	0	0	−1	0	*Radix paeoniae*	0	0	0	0	0	0
*Radix sanguisorbae*	−1.5	−1	−1.5	−1	−0.5	0	*Radix sanguisorbae*	−1.6	−1.5	0	0	−0.5	0
*Radix lithospermi*	0	−1.2	−2	0	−1	0	*Radix lithospermi*	0	0	0	0	0	0
*Scrophulariae*	−2	+1.5	0	0	0	0	*Scrophulariae*	−0.5	0	−1	0	0	0
*Smoked plum*	a	a	a	a	a	a	*Smoked plum*	a	a	a	a	a	a
*Taraxacum mongolicum*	0	0	0	0	0	0	*Taraxacum mongolicum*	0	+2	0	0	0	0
*Tripterygium*	−1.5	0	−2.5	0	0	0	*Tripterygium*	0	0	−2	0	−2.5	0

Note: herbal extracts were used at 1.6 mg/mL. The values shown represent maximal inhibition (fold changes between experiments with and without herb extract). “+” represents induction of gene expression by herb extracts; “−” represents inhibition of biofilm gene expression by herb extracts; “0” represents no effect of biofilm gene expression by herb extracts; “a” represents inhibition of the growth of bacteria. Numbers represent fold changes at the time points where maximal expression was reached in the absence of herbal extract.

**Table 4:** The transcriptional levels of selected genes in PAO1 and PAO1 treated with *H. patriniae*.

Gene	Relative mRNA level determined by ΔCt calculation		
	Without *H. patriniae*	With *H. patriniae*	*P* value
*algU*	1.00	0.192	0.014
*algA*	1.00	0.090	0.011
*pslM*	1.00	0.695	0.043
*bdlA*	1.00	0.412	0.036

Note: the mRNA level of each gene was normalized to that of *proC*. The values shown represent the mean of three different tests.
